# Decoding Mycoplasma Nucleases: Biological Functions and Pathogenesis

**DOI:** 10.3390/toxins17050215

**Published:** 2025-04-24

**Authors:** Xinchao Yi, Ying Huang, Xinru Li, Hao Xu, Chang Liu, Chao Li, Qianrui Zeng, Haodang Luo, Zufeng Ye, Jun He, Xiaoxing You

**Affiliations:** 1 Department of Clinical Laboratory, The Affiliated Nanhua Hospital, Hengyang Medical College, University of South China, Hengyang 421002, China; 2 Hunan Provincial Key Laboratory for Special Pathogens Prevention and Control, Institute of Pathogenic Biology, Hengyang Medical College, University of South China, Hengyang 421001, China; 3 Department of Clinical Laboratory, The Second Affiliated Hospital, Hengyang Medical College, University of South China, Hengyang 421001, China

**Keywords:** mycoplasmas, nuclease, cell invasion, intracellular survival

## Abstract

Nucleases are critical metabolic enzymes expressed by mycoplasmas to acquire nucleic acid precursors from the host for their parasitic existence. Certain nucleases, either membrane-bound or secreted, not only contribute to the growth of mycoplasmas but also serve as key virulence factors due to their unique spatial structures and physiological activity. The pathogenesis includes, but is not limited to, degradation of host DNA and RNA, leading to disruptions of nucleic acid metabolism and the induction of host cell apoptosis; degradation of neutrophil extracellular traps (NETs), allowing escape from neutrophil-mediated killing; and upregulation of inflammatory molecules to modulate the immune response of the host. Understanding the biological functions of nucleases is essential for gaining deeper insights into the virulence and immune evasion strategies of mycoplasmas, which can inform the development of novel approaches for the prevention, diagnosis, and treatment of mycoplasma infections.

## 1. Introduction

*Mycoplasma*, a class of *Mollicutes* bacterial genus lacking cell walls, is among the tiniest self-replicating organisms. It can be isolated from diverse hosts, including humans, insects, and plants [1]. The small size of the mycoplasma genome and the restricted number of encoded proteins limit its biosynthesis capacity, requiring it to source nucleic acid precursors from host organisms to sustain survival and proliferation [2,3,4], with nucleases being essential in this process. Nucleases are a class of hydrolase that catalyze the hydrolysis of phosphodiester bonds in nucleic acids, and their discovery, which dates back to 1903, pertains to enzymes involved in the degradation of nucleic acids [5], when Araki first observed their activity, and the term “nuclease” was coined by Iwanoff [6]. In 1964, the first secreted nuclease was identified in the supernatant of the logarithmic growth medium of multiple mycoplasmas [7]. Subsequently, membrane-bound nucleases were identified in succession, leading to a novel and profound stage in the understanding of mycoplasmal synthetic biology [8]. With subsequent studies, we have found that the functions of *Mycoplasma* nucleases are significantly more complex than previously expected. In many cases, nucleases serve as tools for acquiring nutrients and act as important virulence factors.

Over the past few decades, studies have predominantly concentrated on elucidating the biological properties of nucleases. However, our comprehension of their deleterious effects on the host and immune system has remained notably limited. Due to the limitations of previous *Mycoplasma* gene editing techniques, the acquisition of stable mutant strains has been largely hindered, resulting in much of the literature on nucleases being primarily based on in vitro studies. During the uptake of nucleotide precursors by mycoplasmas for their growth and proliferation, the synergistic effect between specific nucleases and ATP-binding cassette (ABC) transporters is pivotal [9,10]. Some nuclease genes and ABC transporter genes form operon structures in the genome and are co-expressed to achieve efficient uptake of nucleotide precursors [11]. Through persistent investigative efforts, it has become increasingly evident that nucleases serve as significant virulence factors in mycoplasmas [12]. These nucleases not only influence host cell activity but also exert substantial effects on the immune system. The mechanisms underlying these impacts include the degradation of host cell nucleic acids by nucleases, which supply nucleotide precursors for mycoplasmas to synthesize their nutrients. This degradation of DNA and RNA within host cells disrupts nucleic acid metabolism and induces apoptosis. Furthermore, nucleases modulate the expression of multiple inflammatory factors, thereby triggering immune damage in a complex immunological process. Additionally, nucleases play a key role in the degradation of neutrophil extracellular traps (NETs), enabling mycoplasmas to evade neutrophil-mediated killing. Therefore, understanding the pathogenic mechanisms of nucleases offers crucial insights into the molecular interaction between these nucleases in mycoplasmas and host proteins, shedding light on how they contribute to disease and guiding the development of effective preventive strategies.

## 2. Characteristic Domain of *Mycoplasma* Nuclease

Nucleases play a crucial role in the pathogenic mechanisms of Mycoplasma. Although many *Mycoplasma* nucleases share high homology [13], comparative genomic analysis has revealed the presence of various conserved sequences within *Mycoplasma* nucleases [14,15].

### 2.1. TNASE_3 Domain

**Figure 1:**
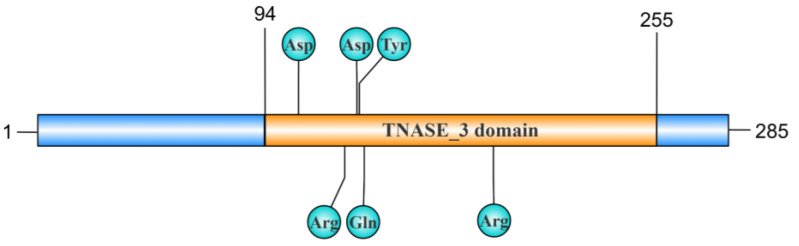
Schematic diagram of the TNASE_3 domain in the mhp379 nuclease. The mhp379 nuclease consists of 285 amino acids, with the TNASE_3 domain spanning from the 94th to the 255th amino acids. Key amino acid sites are marked in the figure. The Ca^2^⁺-binding sites are located at Asp108, Asp132, and Tyr133, while the catalytic sites are Arg127, Gln135, and Arg188. These catalytic sites play a central role in the digestion of nucleic acid substrates by mhp379. This figure was generated using the IBS 2.0 platform.

The thermonuclease domain profile (TNASE_3), originally described in the thermonuclease of *Staphylococcus aureus*, is a highly conserved sequence with several catalytic sites involved in nuclease activity and Ca^2+^ binding [16]. The mhp379 gene in *M. hyopneumoniae* encodes a mature peptide of 285 amino acids, with the TNASE_3 domain located between residues 94 and 255 showing similarities with other nucleases. This domain is conserved in all mhp379 homologues of mycoplasmas, belonging to the thermonuclease cluster of orthologous proteins COG1525 [13]. Interestingly, the aspartate (Asp), Asp, and tyrosine (Tyr) located at positions 108, 132, and 133 in mhp379 are strictly conserved and are reported to be involved in the binding of Ca^2+^ [13]. Additionally, the arginine (Arg), glutamate (Glu), and Arg, located at positions 127, 135, and 188, respectively, are also strictly conserved and constitute the active catalytic sites [13] (Figure 1). The TNASE_3 domain is also present in MG_186, a Ca^2+^-dependent nuclease from *M. genitalium* in which amino acids 44 to 200 exhibit significant homology with the thermonuclease domain profile [17]. The conserved amino acid residues in MG_186 associated with Ca^2+^ binding include Asp (D57, D77) and Tyr (T78), while Arg (R72, R126) and Glu (E80) are also conserved and form part of the active catalytic sites [17]. In *M. pneumoniae*, Mpn133, which shares the highest homology with MG_186, is a nuclease with a similar conserved domain [18]. As expected, these enzymes are Ca^2+^-dependent [18]. Similarly, in other mycoplasma species, the TNASE_3 domain exists in MBOVPG45_0089 and MBOVPG45_0310, two nucleases of *M. bovis* [19]. The TNASE_3 domain not only directly determines the Ca^2+^ of nuclease binding activity but also plays a crucial role in the mycoplasma infection process [20]. For instance, TNASE_3 is essential for MbovNase binding and internalization within cells, as evidenced by the fact that TNASE_3-region-mutant MbovNase is deficient in enzymatic activity, cellular internalization, and nuclear translocation [20].

### 2.2. EEP Domain

**Figure 2:**
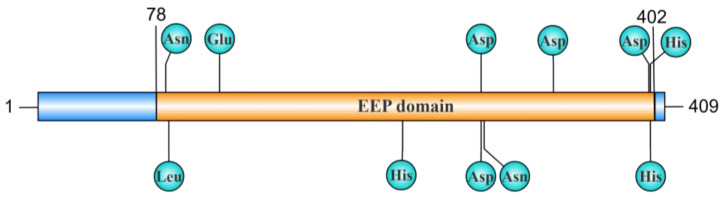
Schematic diagram of the EEP domain in MBOVPG45_0215 nuclease. The MBOVPG45_0215 nuclease is composed of 409 amino acids and contains an EEP domain, which extends from the 78th to the 402nd amino acids. The key amino acid sites are labeled in the figure. Asn84, Glu119, Asp289, Asp336, Asp398, and His399 are involved in Mg^2^⁺ binding, providing the necessary conditions for the protein to exert its nuclease activity. Leu86, His238, Asp289, Asn291, and His399 form a putative phosphate-binding site, influencing the binding of the protein to substrates and catalytic reactions. This figure was created using the IBS 2.0 platform.

Exonuclease–endonuclease–phosphatase (EEP) is a multifunctional enzyme domain existing in a variety of nucleases and playing an important role in nucleic acid metabolism [21]. Employing an HMM-based approach by Saira Mian et al., it was found that the EEP-containing family in eukaryotes and bacteria includes Mg^2+^-dependent endonucleases (L1-EN, DNaseI, APE1, APE2), exonucleases (ExoIII, REX1, REX2), and phosphatases of lipid second messengers (I5PP) [22]. The crystal structures reveal that the central core of EEP domain-containing proteins in humans, yeast, and Schizosaccharomyces pombe is composed of β-sheets surrounded by α-helices, forming a typical α/β sandwich structure that contributes to endonuclease–exonuclease–phosphatase activity [23]. The catalytic site of the EEP domain coordinates metal ions with a single Glu and three Asp residues. This coordination is crucial for its enzymatic activity, as substituting any of these amino acids disrupts its efficacy [22]. The EEP domain is widely present in various nucleases across mycoplasmas, including MBOVPG45_0215 and its homologs in *M. bovis*, MYPU_6930 in *M. pulmonis*, and Mpn491 in *M. pneumoniae* [24] (Figure 2).

### 2.3. EKS Region

**Figure 3:**
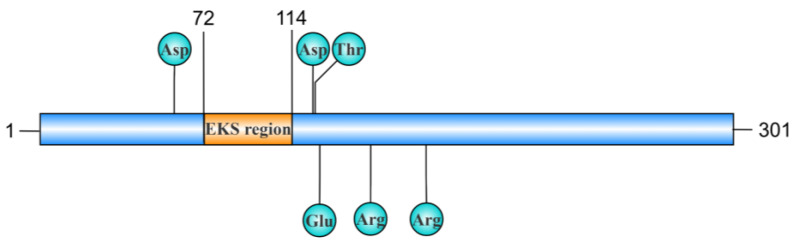
Schematic diagram of the EKS region in Mpn133 nuclease. The Mpn133 nuclease comprises 301 amino acids, and the EKS region is located between the 72nd and 110th amino acids. The Mpn133 with the EKS region deleted (rMpn133^Δ72−110^) has been shown to lose its ability to bind to and internalize into A549 cells. Key amino acid sites are marked in the figure; specifically, Arg114, Arg168, and Glu122 are essential for the nuclease activity of Mpn133, while Asp59, Asp119, and Thr120 are involved in calcium binding, which is crucial for maintaining the Ca^2^⁺-dependent nuclease activity of the protein. This figure was generated using the IBS 2.0 platform.

In 2010, a distinctive region rich in glutamic acid, lysine, and serine (EKS region) was identified and characterized for its essential role in nuclease binding, internalization, and nuclear localization within host cells [18]. One example is Mpn133, a Ca^2+^-dependent cytotoxic nuclease found in *M. pneumoniae*, which possesses a unique EKS region (72–110) that is not necessary for its nuclease activity but plays a critical role in the binding and internalization of airway cells [18]. Mpn133, with a mutant EKS region, cannot bind to and enter cells (Figure 3). This can be further supported by the fact that MG_186 in *M. genitalium,* which lacks the EKS region, failed to bind to and internalize into human endometrial cells [18]. Curiously, Xu et al. found that the MGA_0676 of *M. gallisepticum* is capable of robustly invading DF-1 cells and localizing to the nucleus even in the absence of the EKS region [14]. In addition, Mpn491 of *M. pneumoniae* features a distinct region spanning residues 175 to 218, where the amino acid composition bears more than 70% similarity to EKS [24]. However, their precise function remains elusive [19]. Since these nucleases are biologically membrane-bound, the exact function in the internalization of host cells remains controversial. So far, the EKS structure has not been studied in other mycoplasmas.

### 2.4. YqaJ Domain

**Figure 4:**
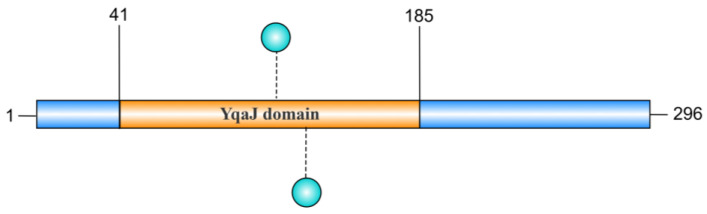
Schematic diagram of the YqaJ domain in MbovP701 nuclease. The MbovP701 nuclease comprises 296 amino acids, and the YqaJ domain is positioned between the 41st and 185th amino acids. The MbovP701 with the YqaJ domain deleted (rMbovP701^Δ41−185^) loses its ability to degrade double-stranded DNA (dsDNA). This figure was produced using the IBS 2.0 platform.

The YqaJ domain is hypothesized to adopt a conserved tertiary structure that facilitates specific nucleic acid recognition and binding. YqaJ-containing proteins (e.g., MbovP701) demonstrate critical functional importance in mycoplasma pathogenesis. Biochemical analyses reveal that the YqaJ domain of MbovP701 (residues 41–185) confers 5′→3′ exonuclease activity, enabling processive degradation of linear double-stranded DNA (dsDNA) substrates [25]. This enzymatic domain exhibits broad substrate specificity, with demonstrated activity against single-stranded DNA (ssDNA), RNA, and supercoiled plasmid DNA. Studies on the truncated mutant rMbovP701^Δ41–185^ have confirmed the indispensability of the domain. Deletion of this region completely abolishes the exonuclease capacity [25]. Although the key amino acid sites within the YqaJ region have not been clearly defined in the current literature, it is speculated that there may be amino acid sites in the YqaJ domain that bind to Mg^2^⁺ and Mn^2^⁺ to regulate enzyme activity; however, there is a lack of direct evidence (Figure 4). These findings establish the YqaJ domain as an essential catalytic module mediating nucleolytic function in mycoplasma proteins [25]. From a pathophysiological perspective, this nuclease activity facilitates microbial nutrient acquisition through efficient nucleic acid catabolism, thereby supporting mycoplasma survival in host microenvironments.

## 3. Biochemical Characterization of Nuclease Activity

Nucleases can be categorized into exonucleases, which cleave nucleic acids from the 5′ or 3′ end, and endonucleases, which break internal phosphodiester bonds without needing a free DNA end [26]. This cleavage mechanism operates via general acid–base catalysis, where the general base deprotonates and activates the nucleophile, while the general acid aids in product formation by protonating the leaving group. Due to the inherent stability of phosphodiester linkages in nucleic acids, the cleavage reaction typically follows an associative SN2 pathway [27]. Mycoplasmas are capable of utilizing undegraded DNA and RNA through membrane-associated nucleases and secretory extracellular nucleases [28]. For example, Bendjennat et al. demonstrated the ability of *M. penetrans* to degrade chromosomal DNA, which highlights the remarkable endonuclease activity [29]. In a similar vein, Paddenberg et al. confirmed the endonuclease function of nucleases from *M. hyorhinis* by showing their capacity to induce DNA laddering [30]. Additionally, the mhp379 nuclease of *M. hyopneumoniae* has been shown to selectively cleave specific DNA sequences, indicating sequence-specific nuclease activity. Nonetheless, the activity of these nucleases is modulated by various factors, such as metal ions, temperature, pH, and substrate structure. These factors, to varying degrees, enable fine-tuning of nuclease activity, allowing mycoplasmas to thrive in complex parasitic environments [13] (Table 1).

### 3.1. Metal Ions

Most *Mycoplasma* nucleases exhibit maximum activity in reaction buffers containing both Ca^2+^ and Mg^2+^, as demonstrated by nucleases from *M. pulmonis*, *M. penetrans*, and *M. hyorhinis* [8,28,31]. For instance, the activity of these ion-sensitive nucleases can be notably increased in the presence of 1 mmol/L Ca^2^⁺, which greatly enhances their nuclease function [13]. However, higher Ca^2+^ concentrations may have a negative impact on their catalytic activity. Similarly, an appropriate Mg^2+^ concentration (0.1 mmol/L) also enhances the activity of MHO_0730 (*M. hominis*), whereas at 0.5 mmol/L, it begins to manifest inhibitory effects [32]. Interestingly, MHO_0730 demonstrates reduced sensitivity to Mg^2^⁺ compared to Ca^2^⁺ [32]. However, unique ion selectivity is exhibited by certain *Mycoplasma* nucleases, which exclusively depend on Ca^2^⁺ and do not require Mg^2^⁺ for their activity. Notable examples include mhp379 (*M. hyopneumoniae*) and MGA_0676 (*M. gallisepticum*) [13,14]. In vitro experiments indicate that mhp379 exhibits optimal nuclease activity when an appropriate concentration of Ca^2^⁺ (approximately 15 mmol/L) is present [13]. Fluctuations in Ca^2^⁺ concentration can significantly impact catalytic efficiency, as deviations from the optimal range result in a marked decrease in enzymatic activity [33]. Consistent with expectations, Mg^2+^ does not significantly affect mhp379 activity under optimal pH and Ca^2+^ concentration conditions [13]. MGA_0676 also shows a similar Ca^2+^-independent activity, as evidenced by degradation of plasmid DNA in vitro [14]. Interestingly, adding Mg^2+^ or Cu^2+^ inhibits this activity, further confirming that the enzymatic activity of MGA_0676 relies specifically on Ca^2+^ rather than other divalent metal ions [14]. Such ion-specific adaptations likely exploit host physiological conditions. For example, intracellular Ca^2^⁺ elevation during apoptosis may activate Mhp379 to degrade host DNA, facilitating nucleotide scavenging.

Given the significant influence of metal ions on nuclease activity, it can be inferred that chelating agents targeting Ca^2^⁺ or Mg^2^⁺ ions may effectively inhibit nuclease function. Notably, Pollack and Hoffmann demonstrated that the endonuclease activity of all *Mollicutes* was entirely suppressed at an EDTA concentration of 20 mmol/L [31]. This finding was corroborated by Bendjennat et al., who showed that EDTA and EGTA substantially inhibit nuclease activity in *M. penetrans* P40, abolishing Ca^2^⁺-mediated activation [29,34]. Similar inhibition is observed in MG_186 (*M. genitalium*), Mpn491 (*M. pneumoniae*), and MAG_5040 (*M. agalactiae*) [17,35]. These results highlight chelators as potential therapeutics that disrupt mycoplasma nutrient acquisition and immune evasion.

### 3.2. Temperature

Temperature critically regulates *Mycoplasma* nuclease activity. Mhp379 (*M. hyopneumoniae*) is most stable between 35 °C and 40 °C, while MGA_0676 (*M. gallisepticum*) exhibits peak activity at 37–49 °C [14]. Similarly, Mpn133 (*M. pneumoniae*) functions optimally at 42 °C, aligning with human respiratory tract temperatures [18]. Activity declines sharply beyond 60 °C, as observed for MHO_0730 (*M. hominis*) [32], indicating evolutionary constraints to maintain function within host physiological ranges. These thermal adaptations ensure enzymatic efficiency during infection. For instance, Mpn133 retains activity during host febrile responses, enabling sustained DNA degradation and immune evasion [18,35]. Collectively, the ion dependencies and temperature optima of *Mycoplasma* nucleases are not merely biochemical traits but adaptive strategies for exploiting host microenvironments, degrading nucleic acids, and evading immune defenses.

**Table 1:** Biological characteristics of the defined *Mycoplasma* nucleases.

Mycoplasma	Nuclease	Amino Acids	Cellular Location	Enzyme Activity	Required Divalent Cation	Substrate	Domain	Optimal Reaction Conditions	UniprotAccession Number	Reference
*M. agalactiae*	MAG_5040	390	Membrane-associated	Endonuclease/exonuclease	Mg^2+^	ssDNA, dsDNA, RNA, plasmid	TNASE_3	Temperature: 37–45 °C, pH: 6–9	A5IYU3	[35]
*M. bovis*	MBOVPG45_0215	409	Membrane-associated	Endonuclease/exonuclease	Ca^2+^, Mg^2+^	dsDNA, plasmid	EEP	Temperature: 37–45 °C, pH: 6–9	A0A454APR1	[19]
*M. bovis*	MbovNase	389	Membrane-associated and secretory	Endonuclease	Ca^2+^	dsDNA, RNA, plasmid	TNASE_3	Temperature: 22–65 °C	Undefined	[20]
*M. bovis*	MbovP701	296	Undefined	Exonuclease	Mg^2+^, Mn^2+^	dsDNA, ssDNA, RNA, plasmid	YqaJ	Temperature: 43 °C, pH: 8.3	Undefined	[25]
*M. hominis*	MHO_0730	321	Membrane-associated	Endonuclease/exonuclease	Ca^2+^	ssDNA, dsDNA, RNA, plasmid	TNASE_3	Undefined	D1J7L0	[32]
*M. pneumoniae*	Mpn133	301	Membrane-associated	Endonuclease	Ca^2+^	ssDNA, dsDNA, RNA, plasmid	TNASE_3EKS	Temperature: 37–49 °C, pH: 8.5	P75265	[18]
*M. pneumoniae*	Mpn491	474	Secretory	DNAse	Mg^2+^	DNA	EEP	Undefined	P75295	[24]
*M. genitalium*	MG_186	250	Membrane-associated	Endonuclease/exonuclease	Ca^2+^	ssDNA, dsDNA, RNA, plasmid	TNASE_3	Temperature: 37–55 °C, pH: 8.3	P47432	[17]
*M. hyopneumoniae*	Mhp379	310	Membrane-associated	Endonuclease/exonuclease	Ca^2+^	ssDNA, dsDNA, RNA, plasmid	TNASE_3	Temperature: 37–45 °C, pH: 8.8	Q600S5	[13]
*M. hyopneumoniae*	Mhp597	377	Secretory	Endonuclease/exonuclease	Ca^2+^, Mg^2+^	ssDNA, dsDNA, RNA, plasmid	Undefined	Undefined	Q5ZZW0	[36]
*M. gallisepticum*	MGA_0676	276	Membrane-associated	Endonuclease/exonuclease	Ca^2+^	ssDNA, dsDNA, RNA, plasmid	TNASE_3	Temperature: 37–49 °C, pH: 8.3	Q7NC48	[14]
*M. meleagridis*	Mm19	646	Membrane-associated	Endonuclease/exonuclease	Mg^2+^	ssDNA, dsDNA, RNA, plasmid	Undefined	Undefined	A0A0U1YYX1	[37]
*M. pulmonis*	MnuA	470	Membrane-associated	Undefined	Undefined	DNA	Undefined	Undefined	Q50321	[38]
*M. penetrans*	P40	Undefined	Membrane-associated	Endonuclease/exonuclease	Ca^2+^, Mg^2+^	ssDNA, dsDNA, RNA, plasmid	Undefined	Temperature: 37 °C, pH: 7–8	Undefined	[34]

## 4. Mechanisms Underlying Nuclease Activity in *Mycoplasma* Pathogenesis

**Figure 5:**
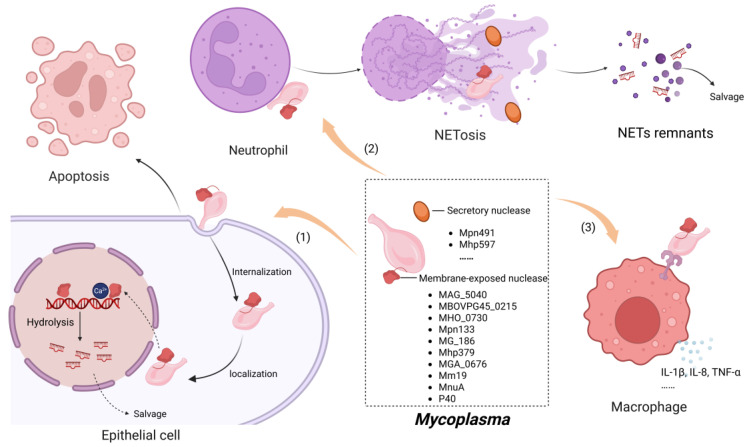
Nucleases are important virulence factors of mycoplasma. They aid mycoplasma metabolism by degrading host nucleotides into precursors for nucleic acid synthesis. (**1**) Once internalized into host cells, they fragment host DNA, causing cytotoxic damage. (**2**) They can degrade NETs, helping mycoplasmas evade neutrophil-mediated killing, and take up nucleic acid precursors for survival. (**3**) They also affect the host’s immune response; certain nucleases induce inflammatory mediator expression and cytokine production. This figure was created using BioRender.com.

Nucleases play a crucial role in the pathogenic mechanisms of mycoplasmas. Although many *Mycoplasma* nucleases share high homology [13], comparative genomic analysis has revealed the presence of various conserved sequences within *Mycoplasma* nucleases [14,15] (Figure 5).

### 4.1. Induction of Host Cell Apoptosis

Apoptosis is a process of programmed cell death that occurs spontaneously. Physiological cell apoptosis plays an important role in the occurrence and development of living organisms. In contrast, pathological apoptosis disrupts metabolic processes and undermines the integrity of normal physiological functions [39]. Apoptosis is a common occurrence in bacterial infections, often triggered by direct interactions between virulence factors and host cells, or through the modulation of specific intracellular signaling pathways [40]. Mycoplasmas have been reported to cause both pro-apoptosis and anti-apoptosis, with outcomes varying based on specific conditions. Recent reports have revealed that *Mycoplasma* nucleases exhibit significant toxicity towards host cells, such as apoptosis or necrosis, an effect that may be partially attributable to nuclease activity [41].

Nucleases play a crucial role as pathogenic factors in mycoplasma infection. Membrane-associated nucleases were reported to exert a dose-dependent reduction in cell viability after binding to host cell membranes in an in vitro study [20]. Upon internalization and subsequent localization within the nucleus, nucleases cleave host DNA into nucleosome-sized fragments. This enzymatic activity results in the degradation of nucleosomes and the condensation of chromatin, processes that collectively culminate in the induction of apoptosis [34]. For instance, incubating the P40 nuclease of *M. penetrans* with CEM or Molt-4 lymphocytic cell lines results in dose-dependent internalization and localization to both the cell membrane and the intracellular compartments. Radiolabeling assays and FITC labeling reveal that the binding process peaks after 15 min, followed by a significant decrease, eventually reaching a steady-state equilibrium. This internalization ultimately leads to apoptosis-like cell death, characterized by plasma membrane blebbing and cytoplasmic shrinkage [34]. Although the exact mechanism underlying nuclease binding and internalization is not fully understood, the EKS sequence seems to play a crucial role since deleting the EKS sequence significantly impairs the internalization process, as demonstrated in the Mpn133 nuclease of *M. pneumoniae*, where removal of the EKS region abolishes both its binding to and internalization by the host cells [18]. However, deletion of the TNASE_3 region does not influence the entry of MGA_0676 from *M. gallisepticum* into DF-1 cells; instead, it reduces the binding affinity of MGA_0676 to the nucleus, resulting in its predominant localization within the cytoplasm [14]. Similarly, the TNASE_3 region of *M. bovis* MbovNase is crucial for cytotoxicity, as the loss of this region leads to deficiencies in nuclease activity, cellular binding, internalization, and nuclear translocation [42].

Internalized nucleases may exert their cytotoxic effects by compromising the structural integrity of DNA and RNA [34]. For instance, incubation of the human T-lymphocytic cell line CEM-13 with recombinant P40 nuclease for 24 h results in internucleosomal DNA degradation, which subsequently leads to plasma membrane blebbing and cytoplasmic shrinkage—hallmarks of programmed cell death [34]. Similarly, internalized Mpn133 triggers deleterious effects in respiratory cells, including reduced viability and apoptotic alterations in phosphatidylserine [18]. Unlike P40 nuclease, the cellular damage observed with Mpn133 does not involve detectable DNA degradation [18]. This difference may be attributed to the direct interaction of Mpn133 with host DNA and RNA, which bypasses internucleosomal fragmentation and inhibits the apoptosis-mediated cell death typically driven by intracellular enzyme activation. Similarly, other membrane-associated nucleases, such as mhp379 in *M. hyopneumoniae* and MG186 in *M. genitalium*, have been shown to internalize into cells, subsequently exerting cytotoxic effects and inducing cell death [13,17]. Physiologically, the free nucleic acids released during apoptotic cell death undoubtedly facilitate uptake by mycoplasmas, thus continuously supplying essential raw materials for their growth [43].

### 4.2. Modulation of Inflammation-Related Molecule Expression by Nucleases

Most nucleases are found in either membrane-bound or secreted forms. This observation prompts speculation regarding the potential pro-inflammatory activity of membrane-anchored nucleases, akin to other membrane-associated lipoproteins, which are considered pro-inflammatory factors for mycoplasmas. Despite the prevalence of unresolved questions within this domain, recent research has identified Mhp597, a nuclease derived from *M. hyopneumoniae*, with significant pro-inflammatory activity [36]. Specifically, Mhp597 upregulates inflammatory cytokines such as IL-1β, IL-8, and TNF-α in porcine alveolar macrophages (PAMs) at both the mRNA and the protein level. Toll-like receptor 4 (TLR4) seems to be responsible for sensing rMhp597 and initiating the inflammatory signal cascade. Further studies revealed that the MyD88-mediated pathway, rather than TRIF, is critical for Mhp597-induced cytokine production [36]. This is intriguing because mycoplasma membrane proteins are typically recognized by TLR2, whereas TLR4 mainly senses LPS or lipids [36]. The acquisition of recombinant Mhp597 usually necessitates prokaryotic expression systems, which inherently carry a risk of endotoxin contamination. Consequently, the assessment of endotoxin levels is crucial when investigating the pro-inflammatory properties of these recombinant proteins. In the study conducted by Li et al., the researchers quantified the endotoxin levels in recombinant Mhp597 and preliminarily excluded the possibility of endotoxin contamination [36]. This finding suggests that the interaction between TLR4 and Mhp597 is likely to result from the specific spatial conformation of Mhp597, a hypothesis corroborated by previous studies [44]. Interestingly, the recombinant rMhp597δ^315−377^, which lacks nuclease activity, continues to exhibit pro-inflammatory properties, suggesting that the pro-inflammatory activity is independent of the nuclease activity but relies on direct interaction with porcine alveolar macrophage cells (PAMs). Unfortunately, the inability to generate an Mhp597-deficient strain poses substantial challenges for directly investigating the activity of Mhp597 [36]. A critical factor is that Mhp597 plays an indispensable role in the vital activities of *Mycoplasma*. Recent research has revealed that Mhp597 interacts with Vim, enhancing Vim protein expression. This interaction inhibits the phosphorylation of TBK1 and IRF3, decreases the release of IFN-I, and facilitates the proliferation and persistent infection of *M. hyopneumoniae* within host cells. Specifically, amino acid residues in Mhp597, such as Lys317, are critical for its binding to Vim. Site mutations at these positions significantly influence the binding affinity between Mhp597 and Vim [45]. The nuclease and pro-inflammatory activities of Mhp597 further underscore the adaptive strategies employed by mycoplasmas to utilize its limited genome for functional diversification [36].

### 4.3. The Evasion of Mycoplasmas from Neutrophil Extracellular Traps Facilitated by Nucleases

Upon infection, the host promptly initiates an innate immune response characterized by the localized release of chemokines to attract neutrophils, facilitating their rapid migration from the bloodstream to the infection site [46]. Following this accumulation, neutrophils release reactive oxygen species (ROS) and myeloperoxidase for the purpose of combating and clearing the invasion of pathogens [47]. More significantly, neutrophils are capable of releasing chromatin, which then forms a DNA–protein reticular structure, referred to as NETs, that plays a crucial role in trapping and neutralizing pathogens [48]. This process underscores the importance of neutrophils in adapting and responding effectively to infection [49,50]. Although neutrophils are not typically regarded as the principal effector cells responsible for clearing mycoplasmas, the mechanism through which *Mycoplasma* species evade neutrophil-mediated killing has remained elusive for many years. It was not until 2016 that Zhang et al. showed that mycoplasma-induced NETs were significantly degraded by *M. bovis*-secreted MbovNase [20], which greatly improved understanding of the role of nucleases in immune evasion.

#### 4.3.1. Formation of NETs

NETs exhibit distinctive antimicrobial activity, characterized by the extrusion of chromatin fibers enriched with granule-derived antimicrobial peptides and enzymes from neutrophils [51]. These structures create a complex scaffold of dispersed DNA strands, histone, and various antimicrobial components predominantly from neutrophil particles, including elastin, myeloperoxidase, calcin, and cathepsin [52]. The formation of NETs entails the disintegration of the cell nucleus and membrane, subsequently leading to the amalgamation of chromatin with cytoplasmic and granule proteins to form a mesh-like structure. Although the pathogens that induce NET formation may differ, the process unfolds in a consistent sequence: (1) blurring between heterochromatin and euchromatin, (2) loss of typical lobular nuclear structure, (3) nuclear membrane disintegration with unchanged cytoplasm and organelles, and (4) vesicle formation, granular membrane dissolution, and nuclear, cytoplasmic, and granular component merging [53]. Upon infection, the formation of NETs is effectively induced by the interaction of mycoplasma pathogen-associated molecular patterns (PAMPs) with TLR2, thereby inducing the formation of NETs through a process known as NETosis [54]. Notably, only liposoluble mycoplasma proteins have been demonstrated to effectively trigger NET formation [54,55]. The downstream signaling pathways mediating the formation of NETs via TLR2 are highly complex. Numerous studies have established a close association between NET formation and the production of ROS [56,57]. It seems that ROS generated by NADPH oxidase are of crucial importance in the signaling pathway of NET formation, as the inhibitors of NADPH oxidase can significantly abolish the formation of NETs [47,57,58]. Moreover, myeloperoxidase (MPO) also exerts an influence on the formation of NETs [58]. Furthermore, alterations in chromatin dynamics, citrullination, and calcium signaling are also essential for NET formation [59]. It should be noted that ROS is not indispensable to the formation of NETs, as stimulation of the MnuA mutant *M. bovis* does not lead to increased ROS accumulation, yet it retains the capacity to induce NET formation [60].

#### 4.3.2. Degradation of NETs by Mycoplasma Nuclease

Staphylococcal nuclease (SNase) is capable of evading entrapment and neutralizes NETs by enzymatically degrading the DNA backbone of NETs, diminishing their structural integrity and facilitating their clearance [61]. Given that NETs are composed of DNA originating from the nucleus or mitochondria [62], and *Mycoplasma* nucleases exhibit both exonuclease and endonuclease activities, it is plausible to infer that nucleases also play a role in the degradation of NETs. Indeed, as early as 2016, Zhang et al. demonstrated that the nuclease of *M. bovis* is capable of degrading NETs, with the TNASE_3 domain potentially playing a crucial role [20]. The authors suggested that secreted nucleases, as opposed to membrane-bound nucleases, may provide more favorable conditions for NET degradation. Secreted nucleases can diffuse and assist other bacteria in evading NETs. The study convincingly showed that the Ca^2^⁺-dependency of MbovNase can degrade NETs released in response to the presence of other bacteria, such as *Mannheimia haemolytica* [20].

Furthermore, the Mpn491 nuclease of *M. pneumoniae* is also essential for the bacterium’s defense against NET killing [24]. The Mpn491-deficient strain, which failed to effectively degrade PMA-induced NETs, showed reduced survival in vitro. However, the bacterial load could be markedly restored upon DNase I treatment. In vitro studies also indicated that Mpn491 significantly promotes the degradation of LPS-induced NETs compared to the Mpn491-deficient strain [24]. This evidence emphasizes that Mpn491 plays a crucial role in enabling *M. pneumoniae* to evade the immune response by degrading NETs [24]. Interestingly, the membrane-bound nucleases of *M. hyopneumoniae* can also degrade macrophage extracellular traps (METs), which originate from the human monocytic cell line THP-1 and share structural similarities with NETs, thereby facilitating the necessary nucleotide availability for efficient DNA synthesis [63]. The inhibition of the active site of *M. hyopneumoniae* nuclease by chelating agent and anti-Mhp597 profoundly abrogates the degradation of METs and the transfer of nucleotides, highlighting their crucial role in this process. A 5-ethynyl-2′-deoxyuridine (EdU)-labeling experiment demonstrated that Edu was derived directly from METs rather than from free intracellular nucleotides, indicating that the degradation of NETs by nucleases is another important source of nucleotides for nucleic acid synthesis [63]. Similar activities can also be found in the MnuA from *M. bovis* and Mhp597 from *M. hyopneumoniae*. The degradation of NETs (or METs) by *Mycoplasma* nucleases can effectively reduce the direct damage that NETosis might cause to mycoplasmas [36].

However, it is important to note that NETs possess a wide variety of functions. For instance, they can act as damage-associated molecular patterns (DAMPs), which can trigger and initiate further inflammatory responses in the body [64,65]. In addition, NETs can activate macrophages, leading to the secretion of the pro-inflammatory cytokines IL-8, IL-6, and TNF-α. NETs can also directly activate dendritic cells (DCs) [66], inducing the production of the co-stimulatory molecules CD80 and CD86 and the pro-inflammatory cytokine IL-6. Given these diverse functions of NETs, the destruction of NETs by nucleases may have a far-reaching and profound impact on the immune system, potentially affecting various aspects of immune regulation and response [46].

## 5. Prospects and Conclusions

Nucleases have garnered increasing attention in recent years as a key factor in bacterial growth, metabolism, and pathogenicity. These enzymes, responsible for degrading DNA and RNA, play essential roles in mycoplasmas by contributing to cytotoxicity, inflammation, and immune evasion. Despite significant advances in the study of *Mycoplasma* nucleases, numerous challenges persist in this field.

First, the diversity of *Mycoplasma* nucleases poses a fundamental challenge. Variations in sequence, structure, and function complicate efforts to fully comprehend the prevalence and specificity of these enzymes. The intricate interactions between their diverse functions further complicate the pursuit of a comprehensive understanding. Second, the regulatory mechanisms governing *Mycoplasma* nuclease expression remain poorly understood. Given the close relationship between nuclease structure and function, elucidating their three-dimensional structures and interactions with substrates represents another significant hurdle. Some nucleases have highly flexible domains, leading to multiple conformations upon substrate binding, which complicates structural analysis [13,29]. The degradation of NETs by *Mycoplasma* nucleases has been identified as an immune evasion strategy in recent years [24,42,60]. However, the complexity of these immune evasion strategies and the fine-tuned interaction between bacterial nucleases and the host immune response remain poorly understood, representing an additional layer of complexity.

Although foundational knowledge on nucleases continues to expand, translating these findings into clinical applications remains challenging. For instance, the development of inhibitors targeting bacterial nucleases shows potential for anti-infective therapies [67]. However, the diversity and complexity of *Mycoplasma* nucleases make the design and development of such inhibitors particularly difficult. Additionally, bacterial nucleases may influence antibiotic resistance through various pathways [68], raising concerns about the safety and efficacy of nuclease-targeted treatments.

Future studies should focus on the precise roles of bacterial nucleases in host–pathogen interactions. Clarifying how different nuclease types enable mycoplasma to bypass immune defenses, and how the host adjusts its immune responses to counteract nuclease activity, will deepen understanding of the molecular mechanisms driving mycoplasma infections. Such insights could also provide a foundation for new therapeutic approaches.

Resolving the three-dimensional structures of *Mycoplasma* nucleases remains a priority. Advances in structural biology techniques, such as cryo-electron microscopy, nuclear magnetic resonance (NMR) spectroscopy, and computational modeling, can reveal high-resolution structures. Recent advances in computational tools such as AlphaFold have enabled high-accuracy prediction of protein structures, even for understudied targets [69]. Applying this approach to *Mycoplasma* nucleases (e.g., Mhp379, Mpn133) could resolve their structural basis, such as Ca^2^⁺-binding motifs or flexible regions critical for host interaction. While experimental validation remains essential, AlphaFold models would accelerate functional studies and guide inhibitor design by mapping catalytic pockets. Future collaborations integrating computational and experimental efforts are needed to fully exploit this potential.

Beyond their biological roles, nucleases hold significant potential in biotechnology and synthetic biology. Engineering specific nucleases for DNA/RNA editing and genomic studies offers valuable tools for molecular biology [70,71,72]. Additionally, the specificity and efficiency of bacterial nucleases can be harnessed for precise regulation of biological systems [73,74]. As an important virulence factor, nucleases should also be integrated into vaccine strategies against *Mycoplasma* in the future [75]. These applications not only contribute to fundamental discoveries but also open up avenues for industrial and medical innovation.

Some *Mycoplasma* species encode multiple nucleases [19]. For instance, MBOVPG45 possesses three annotated membrane-bound nucleases: MBOVPG45_0089, MBOVPG45_0215 (mmuA), and MBOVPG45_0310. Sharma et al. demonstrated through transposon mutagenesis that disruption of mmuA abolished ~90% of cellular exonuclease and endonuclease activity, while inactivation of MBOVPG45_0310 had no significant impact. The redundancy of such important activity, as well as the role of each protein, should be considered.

Notably, nuclease secretion has not been experimentally detected in members of the *Mycoplasma mycoides* cluster, as reported by Minion et al., and in silico genomic analyses by Sharma et al. failed to identify homologs of known nucleases [19]. This observation raises intriguing questions regarding the molecular mechanisms underlying nucleic acid metabolism in these pathogens. The apparent absence could stem from methodological limitations in detection techniques or bioinformatics tools, which may overlook divergent enzymatic motifs or non-canonical nuclease families. Alternatively, it may reflect evolutionary adaptations unique to this phylogenetic lineage, such as reliance on host-derived nucleases or alternative pathways for nucleic acid processing.

In summary, the diversity of *Mycoplasma* nucleases, their regulatory mechanisms, structure–function relationships, and complex interplay with host immunity represent key focus areas. As advances in structural biology, molecular biology, and bioinformatics continue, further clarity on nuclease functions is expected, providing new targets and strategies for combating infections. Sustained efforts in this field hold the potential to unlock significant breakthroughs in public health.

## Data Availability

No data were generated or analyzed in this work.
